# Hepatitis E virus (HEV) infection among the Arab population in Northern Israel: an insight into the seroepidemiology and associated risk factors

**DOI:** 10.1017/S0950268824001407

**Published:** 2025-01-13

**Authors:** Orna Mor, Wasef Na’amnih, Rachel Shirazi, Marina Wax, Yael Gozlan, Marah Kassim, Helal Sayid, Ali Omari, Adel Jabbor, Khitam Muhsen, Amir Mari

**Affiliations:** 1 Department of Epidemiology and Preventive Medicine, School of Public Health, Faculty of Medicine, Tel-Aviv University, Tel-Aviv, Israel; 2 Central Virology Laboratory, Ministry of Health, Public Health Services, The Chaim Sheba Medical Center, Tel HaShomer, Ramat-Gan, Israel; 3 Gastroenterology Department, Nazareth Hospital EMMS, Azrieli Faculty of Medicine, Bar Ilan University, Ramat-Gan, Israel; 4 Medical Laboratory, Nazareth Hospital EMMS, Nazareth, Israel

**Keywords:** Age, Hepatitis E virus, immunoglobulin G, lifestyle, seroprevalence, sociodemographic factors

## Abstract

Hepatitis E virus (HEV) is one of the most common causes of viral hepatitis. We examined HEV seroprevalence and associations of sociodemographic and lifestyle characteristics with HEV immunoglobulin G (IgG) seropositivity in the Arab population. A cross-sectional single-centre study was conducted among adults in the Nazareth area during 2022. Blood samples were tested using the Altona Real-Star HEV-RNA and the Wantai IgG assays. Data on sociodemographics, health status, and lifestyle were collected using structured questionnaires.

Overall, 490 individuals (55.9% males) aged 18 − 96 (mean = 53.2, SD = 28.0) were enrolled. HEV IgG seropositivity was estimated at 21.4% (95% CI 17.9–25.3). No samples were HEV-RNA positive. The correlates of HEV IgG seropositivity were older age (prevalence ratio (PR) 1.07, 95% CI 1.04–1.09, *P* < 0.001) and consuming beef frequently (PR 2.81, 95% CI 1.40–5.63, *P* = 0.003). No associations were found between Arab religious groups (Muslim, Christian or Druze, representing different socioeconomic status and dietary habits) or pork consumption and HEV IgG seropositivity. In conclusion, HEV seropositivity was high in the Arab population, and assessing HEV in Ruminants, particularly cows, is warranted.

## Introduction

Hepatitis E virus (HEV) is the most common cause of liver infection in developing countries and is considered an underestimated emerging pathogen in developed countries [1]. There are an estimated 20 million HEV infections annually worldwide [2]. HEV is part of the family *Hepeviridae* that includes enterically-transmitted, small, quasi-enveloped viruses with positive-sense RNA genomes. The *Hepeviridae* family includes two subfamilies: *Parahepevirinae* and *Orthohepevirinae.* The latter can further be divided into four genera: Avihepevirus genus, Chirohepevirus genus, Rocahepevirus genus, and Paslahepevirus genus. HEV belongs to the Paslahepevirus genus [3, 4]. Eight different HEV genotypes are currently recognized [5], of which HEV-1, HEV-2, HEV-3, HEV-4, and HEV-7 have been associated with human infections [6]. HEV-1 and HEV-2 are endemic in developing countries, while HEV-3 and HEV-4 are mainly related to infections in humans in industrialized countries. HEV-1 and HEV-2 cause acute infections only and are mainly transmitted through contaminated water [6]. HEV-3, HEV-4, and HEV-7 generally cause asymptomatic infection; however, they were also linked to both acute and chronic viral hepatitis [7]. HEV-3, HEV-4, and HEV-7 are zoonotic genotypes that infect humans through the consumption of raw or undercooked meat and meat products from domestic or wild animals contaminated with the virus or by direct contact with infected animals. HEV-3 and HEV-4 have a broad host range, are endemic in pig farms, and transmission to humans has been linked mainly to ingestion of undercooked pork or meat. Recently, HEV-3 has also been reported in ruminants, especially in cows, goats, sheep, and buffalos, and was also shown to be spread from pigs to sheep in Mongolia [8]. HEV-7 was identified in dromedary camels and in a single case of chronic viral hepatitis following ingestion of camel-derived meat and milk [9]. Bloodborne transmission of HEV has also been documented [10].

In Israel, sequences of HEV-1, HEV-3, and HEV-7 have been identified [11–16]. However, HEV-1 was the only genotype detected in humans and was linked to travellers returning from developing countries [17]. HEV-3 was found to be endemic in pig farms located in Northern Israel, and antibodies against HEV were detected in all farmworkers exposed to these pigs [10]. However, to our knowledge, HEV-3 has not been detected in Israel in humans. Similarly, a complete HEV-7 sequence was isolated from local dromedary camel blood without any documented HEV-7 infection in humans [10]. Autochthonous rare cases of acute hepatitis, which were positive for HEV, have also been reported, and local circulation of this virus was suggested; however, the infecting genotype remained undefined [12, 17]. In such circumstances, such as with HEV, when infection is mainly silent and acute cases are rarely reported, a sero-survey that assesses factors related to the infection may provide a lead to the correlates of the identified seropositivity.

The pig population in Israel consists of about 120000 pigs at any given moment, and most are bred in farms located in the north of Israel and are used for local pork consumption. However, not all religions allow eating pork and its products. Traditionally, Muslims and Druze Arabs (as well as many Jews) refrain from consuming pig products, while Christian Arabs have no religious restrictions on pork consumption.

Arabs comprise approximately 20% of the population in Israel. The majority are Muslims (72.5%), followed by Druze (14.7%) and Christians (12.8%) [18]. We and others have previously reported a higher rate of seropositivity of HEV immunoglobulin G (IgG) in the Arab population compared to the Jewish population [11, 13, 19]; however, correlates of HEV seropositivity in the Arab population were not thoroughly investigated.

Accordingly, the aims of this study were to assess the seroprevalence of HEV in this population and examine potential correlates of HEV IgG seropositivity. Our underlying hypotheses were that HEV IgG seropositivity might be related to sociodemographic factors (e.g., age, religion, and education), lifestyle (e.g. dietary consumption), and health status.

## Material and methods

### Study design and population

This cross-sectional study was undertaken between March and October 2022 among adults aged 18 years or older attending the gastroenterology unit of the Nazareth Hospital, a 150-bed regional teaching hospital in Nazareth City. This hospital provides healthcare services for the population in the north of Israel. The population in this region comprises Arab and Jewish residents who usually live in separate towns and cities. Based on the Central Bureau of Statistics, about 1.47 million people lived in the north district in 2020, with Arabs being the majority in this region (57%, 805000 residents) [18].

Access to healthcare in Israel is universal following the National Health Insurance Law [20], covering both outpatient and inpatient health services. The Arab towns in the north of Israel have been undergoing ongoing improvement in sanitation infrastructure. All towns and villages are connected to the national piped water system and electricity. However, socio-economic differences between the Christians and Muslims in Israel still exist [21].

### Sample size calculation

The sample size was calculated assuming a 10% HEV seropositivity [9], and 20% prevalence in exposed groups, a 2-sided type-1 error of 5%, and statistical power of 80%; accordingly, the minimal needed sample size was 200 per group (e.g., exposed and unexposed to factors such as pork consumption, Muslims or Christians, total 400) to detect such a difference. We aimed to enrol 500 participants to account for refusals.

### Data and sample collection

Consecutive patients visiting the gastroenterology unit in Nazareth Hospital were offered to participate in the study. Those who agreed and signed a written informed consent were asked to fill out a paper questionnaire in Arabic that was completed anonymously and aimed to collect information on sociodemographics, health status, and lifestyle, including historical and current dietary consumption. Blood samples were collected from each participant by the study nurses and physicians. The main inclusion criterion was the willingness to participate in the study and provide a blood sample. The response rate was ~90%.

### Definition of the study variables

The main dependent variable was HEV seropositivity (seropositive IgG vs. seronegative IgG) as determined by ELISA (see laboratory methods). The independent variables included sociodemographics [(age at the time of the interview, analyzed as a continuous and categorical variable (18–44, 45–64, 65–96, in years), sex, religion (Christians, Muslims or Druze), number of schooling years (a continuous variable), employment status (yes or no), and household density (the number of individuals living in a household divided by the number of the rooms in the household). Cooking habits [frequency of cooking/preparing food at home (4–5 times a week, less often)], methods for preparing food (cooked, fried, barbequed, other), surfaces used for cutting meat (wood, plastic, other). The variables on dietary consumption included the weekly frequency of consumption, in general, of the following: fruit and vegetables (4–5 times vs. less), chicken (2–5 times vs. less), beef (2–5 times vs. less), mutton (2–5 times vs. less), and fish (2–5 times vs. less) consumption of pork (yes or no), camel meat (yes or no), seafood, and/or clams (yes or no). The categories of the food items were determined based on how common the consumption of a certain item is. For example, fruit, vegetables, meat, and fish, which were commonly consumed, were categorized based on weekly consumption, while pork and camel meat, which were less often consumed, were categorized as yes vs. no.

We did not collect information on changes in dietary habits.

Environmental variables included pet ownership (yes or no) and living near farms (yes or no). Health status variables were having a history of hypertension (yes or no), diabetes mellitus (yes or no), heart disease (yes or no), inflammatory bowel disease, stones in the gallbladder, surgery (any, yes or no), and ever having a blood transfusion (yes or no). These variables were selected based on previous studies suggesting that HEV might be transmitted by food and that it may be related to specific environmental and demographic factors [1, 22, 23]. Data on lifestyle habits (current smoking (yes or no), alcohol consumption (yes or no), regular physical activity (yes, no), and symptoms were collected to characterize the study sample.

### Laboratory analysis

Fresh whole blood samples were collected and immediately transferred to the laboratory in the Nazareth hospital. Plasma was separated and stored at −80 °C until transferred frozen to the National HIV and Viral Hepatitis reference laboratory in Tel HaShomer, Ramat-Gan, for laboratory analysis. Overall, 200 μl of plasma was used for RNA extraction using MagLEAD 12gC (PSS, Japan). HEV RNA was assessed with the RealStar HEV kit (Altona Diagnostics GmbH, Hamburg, Germany). The presence of anti-HEV IgG antibodies in serum was measured using the Wantai ELISA kit (Wantai, Biologic Pharmacy Enterprise, Beijing, Republic of China), a test that recognizes human antibodies against all HEV genotypes and was reported to have 97.96% sensitivity and 99.99% specificity compared to commercially available HEV ELISA tests [24]. All assays were performed according to the manufacturer’s instructions and blinded to patients’ background characteristics. All samples with >1.1 S/CO values were considered positive, and all samples with >0.9 S/CO values were recorded as negative. The median IgG S/CO value of the IgG-positive samples was 13.8 (IQR 12.7), and the median S/CO value of the negative samples was 0.005 (IQR 0.03). Three samples with equivocal results (S/CO values between 0.9 and 1.1) were considered seronegative in the analysis. Table 1 in the supplementary section shows all S/CO values. Supplementary Figures 1 and 2 demonstrate the histogram and box plot of the HEV IgG S/Co value distribution.

**Table 1.** Characteristics of the participants by population group

	**Overall** **n=490 ^a^**	**Muslims** **n=235**	**Druze** **n=39**	**Christians** **n=203**	**P value ^b^**
**Age, years, mean (SD)**	53.2 (18.0)	53.3 (17.9)	49.3 (14.7)	57.0 (18.7)	0.335 ^c^
**Number of schooling years, mean (SD)**	11.1 (3.9)	10.3 (4.0)	11.1 (4.6)	11.9 (3.5)	<0.001 ^c^
**Household density, mean (SD)**	1.12 (0.54)	1.18 (0.62)	1.11 (0.43)	1.06 (0.44)	0.084 ^c^
**Age group, years**					0.615
18–44	149 (31.9%)	68 (30.1%)	14 (35.9%)	63 (32.0%)	
45–64	190 (40.3%)	96 (42.5%)	17 (43.6%)	73 (37.0%)	
65–96	133 (28.2%)	62 (27.4%)	8 (20.5%)	61 (31.0%)	
**Sex**					0.008
Males	269 (55.9%)	121 (51.5%)	16 (42.1%)	129 (63.5%)	
Females	212 (44.1%)	114 (49.5%)	22 (57.9%)	74 (36.5%)	
**Employment,** yes	244 (51.4%)	105 (45.3%)	18 (47.4%)	120 (60.0%)	0.008
**Frequency of fruit/vegetable consumption**					0.699
4–5 times a week	393 (83.3%)	195 (84.8%)	33 (84.6%)	162 (81.8%)	
Less than 4 times a week	79 (16.7%)	35 (15.2%)	6 (15.4%)	36 (18.2%)	
**Frequency of chicken consumption**					0.338
2–5 times a week	335 (71.0%)	154 (67.5%)	28 (71.8%)	148 (74.0%)	
Less than 2 times a week	137 (29.0%)	74 (32.5%)	11 (28.2%)	52 (26.0%)	
**Frequency of fish consumption**					0.700
1–5 times a week	243 (52.9%)	116 (52.0%)	23 (59.0%)	100 (51.8%)	
Less than 1times a week	216 (47.1%)	107 (48.0%)	16 (41.0%)	93 (48.2%)	
**Frequency of beef consumption**					0.083
2–5 times a week	108 (23.3%)	44 (19.6%)	8 (20.5%)	56 (28.7%)	
Less than 2 times a week	355 (76.7%)	180 (80.4%)	31 (79.5%)	139 (71.3%)	
**Frequency of mutton consumption**					0.205
2–5 times a week	75 (16.3%)	32 (14.4%)	4 (10.3%)	38 (19.6%)	
Less than 2 times a week	385 (83.7%)	190 (85.6%)	35 (89.7%)	156 (80.4%)	
**Pork consumption,** yes	129 (28.2%)	5 (2.3%)	1 (2.6%)	123 (63.7%)	<0.001
**Camel meat consumption,** yes	10 (2.2%)	2 (0.9%)	0 (0.0%)	8 (4.3%)	0.045
**Seafood/Clams consumption**, yes	125 (27.0%)	20 (8.9%)	8 (20.5%)	95 (49.0%)	<0.001
**Pets ownership,** yes	78 (16.3%)	29 (12.4%)	7 (17.9%)	41 (20.4%)	0.075
**Living near farm animals,** yes	20 (4.2%)	16 (6.8%)	1 (2.6%)	3 (1.5%)	0.019
**Current smoking,** yes	168 (35.6%)	73 (31.5%)	7 (18.9%)	84 (42.4%)	0.006
**Alcohol consumption,** yes	142 (30.0 %)	20 (8.6%)	5 (13.5%)	117 (58.8%)	<0.001
**Hypertension,** yes	163 (35.9%)	86 (37.7%)	12 (31.6%)	65 (35.5%)	0.736
**Diabetes mellitus,** yes	140 (30.8%)	76 (33.2%)	7 (18.4%)	56 (30.6%)	0.188
**Heart disease,** yes	56 (12.4%)	29 (12.7%)	5 (13.5%)	22 (12.1%)	0.964
**Blood transfusion**, ever (yes)	32 (6.9%)	11 (4.8%)	4 (11.1%)	16 (8.1%)	0.220

a The study included 490 participants. Information was missing for some variables in the questionnaires (see supplementary table 1), therefor the numbers do not add-up to 490.

b P value was obtained by the chi-square test

c One Way ANOVA of Variance, SD=standard deviation.

### Statistical analysis

The study sample was described using median and interquartile range (IQR) for continuous variables with skewed distribution. The assumption of the normal distribution was tested by the Kolmogorov–Smirnov test. Differences between Muslims, Druze, and Christians in sociodemographics, health status, environmental factors, and lifestyle were examined using the chi-square test and Fisher exact test where appropriate for categorical variables, the Student’s *t*-test for continuous variables, and Mann-Whitney for variables with skewed distribution. The overall and age-stratified HEV IgG seropositivity and 95% confidence interval (CI) were calculated and expressed in percentages. Differences between HEV IgG antibody seropositive and seronegative individuals in sociodemographic, environmental factors, lifestyle, and health-related factors were examined using the chi-square test and Fisher exact test where appropriate for categorical variables, the Student’s *t*-test for continuous variables when comparing two groups, and one-way analysis of variance (ANOVA) when comparing three groups. Multivariable analysis was performed using generalized linear models with a negative binomial regression model to examine the correlates of HEV IgG seropositivity while adjusting for other variables in the model. The inclusion of variables in the multivariable model was based on our hypothesis of an association between demographics and lifestyle with HEV seroprevalence. Prevalence ratio (PR) and 95% CI were obtained for each variable in the model. A two-sided *P* value<0.05 was considered statistically significant. To handle missing data, we used the complete-case analysis approach.

Data were analyzed using the Statistical Package for the Social Science (SPSS) version 28 (IBM, Armonk, New York, NY, USA).

#### Ethical considerations

All procedures performed in this study were conducted according to the ethical standards of the institutional and national research committee and the 1964 Declaration of Helsinki and its later amendments or comparable ethical standards. The study protocol was approved by the institutional review board of the Nazareth Hospital EMMS (35–33-EMMS, March 2022). All participants signed a written informed consent form.

## Results

### Description of the study sample

Overall, 490 individuals were included in the study. Of these, 477 (97.3%) completed the questionnaire: 235 (49.3%) Muslims, 39 (8.1%) Druze, and 203 (42.6%) Christian Arabs.

The participants’ ages ranged from 18 to 96 years (mean 53.2 years, SD = 18), with 27.1% of the participants being 65–96 years old. Females comprised 43.8% of the sample. The mean number of schooling years was 11.1 (SD = 3.9), and 51.4% of the participants reported being employed. No significant differences were found between the groups in age, household density, frequency of consumption of fruit, vegetables, beef, chicken, or fish, pet ownership, background diseases, and history of blood transfusion. However, the mean number of schooling years was significantly (*P* < 0.001) lower in Muslims (10.3) compared to Druze (11.1) and Christians (11.9). The percentages of females differed between the groups being 49.5%, 56.4%, and 36.5% in Muslims, Druze, and Christians, respectively, *P* = 0.008, and the respective percentages of employed individuals were 44.7%, 46.2%, and 59.1%, *P* = 0.008. The groups significantly differed in some dietary habits, with higher reports on consumption of pork found in Christians compared to Muslims and Druze (63.7%, 2.3%, and 2.6%, respectively, *P* < 0.001), seafood and/or clams (46.8%, 8.5%, and 20.5%, respectively, *P* < 0.001), and alcohol consumption (57.6%, 8.5% and 12.8%, respectively, *P* < 0.001). Consumption of camel meat was uncommon, reported by 10 participants (2.2%), mostly Christians. Living near farm animals was rarely reported and was less common in Christian participants (1.5%) than in Muslim (6.8%) and Druze participants (2.6%), *P* = 0.019 (Table 1).

### Health status and health behaviours of the participants

The main complaints were abdominal pain and heartburn, which were reported frequently (at least 3 times a week) by 264 (53.9%) and 218 (44.5%) of the participants, respectively. Other reports on gastrointestinal symptoms included nausea and vomiting (12.9%), constipation (11.0%) and diarrhea (6.3%). Inflammatory bowel disease was reported by 25 (5.1%) participants, and 10 (2.0%) reported stones in the gallbladder.

About one-third of the study participants reported having a diagnosis of hypertension or diabetes mellitus, and 12.4% reported having a heart disease. Regular physical activity was reported by 22.4% of the participants. Current smoking was reported by 40.9% and 7.1% of the participating men and women, respectively. The age and sex estimates of these conditions with those reported in the general Arab population were similar (Supplementary table 2).

### HEV testing

All samples tested negative for HEV-RNA. Overall, 105 individuals were seropositive for HEV IgG antibody, yielding a seropositivity of 21.4% (95% CI 17.9–25.3). HEV seropositivity increased with age from 2.0% (95% CI 0.4–5.8) in individuals aged 18–44 years to 12.6% (95% CI 8.3–18.2) and 55.6% (95% CI 46.8–64.3) in the age groups 45–64 and 65–96 years, respectively. HEV seropositivity was higher among Muslims 25.9% (95% CI 20.5–32.1) than Druze 12.8% (95% CI 4.3–27.4) and Christians 17.7% (95% CI 12.7–23.7), *P* = 0.044.

### Factors associated with HEV IgG seropositivity

HEV IgG seropositive individuals were significantly older and had a lower median number of schooling years, and the number of employed individuals was lower in that group than the seronegative ones (*P* < 0.001). The percentages of Muslims were significantly higher in the seropositive group (*P* = 0.04) and the percentage of employed individuals was lower compared to the seronegatives (*P* < 0.001). No significant differences were found in HEV seropositivity according to sex (*P* = 0.9) or household density (*P* = 0.8). Assessment of dietary habits revealed that consumption of beef 2–5 times a week was more common in the seropositive vs. seronegative group: 31.4% vs. 19.5% (*P* = 0.01), while the opposite was found for consumption of seafood and/or clams (12.4% vs. 29.1%, *P* < 0.001) which was less commonly reported in the seropositive group. No significant associations were found between other dietary habits, including pork consumption, and HEV IgG positivity (Table 2). Pet ownership was less common in the seronegative group (*P* = 0.04) but living near farm animals was not different between the two groups (*P* = 0.7). Blood transfusions (*P* = 0.03) and comorbidity with diabetes, heart disease, and hypertension (*P* < 0.001) were reported significantly more often in the seropositive vs. the seronegative group.

**Table 2.** Factors associated with HEV IgG seroprevalence^a^

	Seropositive *n* = 105	Seronegative *n* = 385	*P* value^b^
**Age, years, mean (SD)**	71.2 (12.1)	48.3 (16.1)	<0.001^c^
**Schooling years, mean (SD)**	8.5 (4.5)	11.7 (3.5)	<0.001^c^
**Household density, mean (SD)**	1.11 (0.48)	1.13 (0.55)	0.8^c^
**Age group**			<0.001
18–44	3 (2.9%)	146 (37.9%)	
45–64	24 (22.9%)	166 (43.1%)	
65–96	74 (70.5%)	59 (15.3%)	
Missing	4 (3.8%)	14 (3.6%)	
**Sex**			0.9
Males	57 (54.3%)	212 (55.1%)	
Females	46 (43.8%)	166 (43.1%)	
Missing	2 (1.9%)	7 (1.8%)	
**Religion**			0.04
Muslims	61 (58.1%)	174 (45.2%)	
Druze	5 (4.8%)	34 (8.8%)	
Christian	36 (34.3%)	167 (43.4%)	
Missing	3 (2.9%)	10 (2.6%)	
**Employment,** yes	24 (22.9%)	220 (57.1%)	<0.001
Missing	3 (2.9%)	12 (3.1%)	
**Frequency of preparing/cooking food at home**			0.2
4–5 times a week	66 (62.9%)	218 (56.8%)	
Less than 4 times a week	35 (33.3%)	150 (39.1%)	
Missing	4 (3.8%)	16 (4.2%)	
**Methods of preparing/cooking the food**			0.7
Fried	32 (30.5%)	110 (28.6%)	
Other	70 (66.7%)	259 (67.3%)	
Missing	3 (2.9%)	16 (4.2%)	
**Surfaces used for cutting meat or chicken**			0.2
Wood	51 (48.6%)	216 (56.1%)	
Plastic	44 (41.9%)	123 (31.9%)	
Other	7 (6.7%)	29 (7.5%)	
Missing	3 (2.9%)	17 (4.4%)	
**Frequency of fruit and vegetable consumption**			0.5
4–5 times a week	88 (83.8%)	305 (79.2%)	
Less than 4 times a week	15 (14.3%)	64 (16.6%)	
Missing	2 (1.9%)	16 (4.2%)	
**Frequency of chicken consumption**			0.7
2–5 times a week	72 (68.6%)	263 (68.3%)	
Less than 2 times a week	31 (29.5%)	106 (27.5%)	
Missing	2 (1.9%)	16 (4.2%)	
**Frequency of fish consumption**			0.4
2–5 times a week	12 (11.4%)	55 (14.3%)	
Less than 2 times a week	86 (81.9%)	306 (79.5%)	
Missing	7 (6.7%)	24 (6.2%)	
**Frequency of beef consumption**			0.01
2–5 times a week	33 (31.4%)	75 (19.5%)	
Less than 2 times a week	69 (65.7%)	286 (74.3%)	
Missing	3 (2.9%)	24 (6.2%)	
**Frequency of mutton consumption**			0.2
2–5 times a week	20 (19.0%)	55 (14.3%)	
Less than 2 times a week	80 (76.2%)	305 (79.2%)	
Missing	5 (4.8%)	25 (6.5%)	
**Pork consumption,** yes	21 (20.0%)	108 (28.1%)	0.08
Missing	6 (5.7%)	27 (7.0%)	
**Camel meat consumption,** yes	1 (1.0%)	9 (2.6%)	0.3
Missing	7 (6.7%)	37 (9.6%)	
**Seafood/Clams consumption,** yes	13 (12.4%)	112 (29.1%)	<0.001
Missing	5 (4.8%)	22 (5.7%)	
**Pets ownership,** yes	10 (9.5%)	68 (17.7%)	0.04
Missing	2 (1.9%)	9 (2.3%)	
**Living near farm animals,** yes	5 (4.8%)	15 (3.9%)	0.7
Missing	2 (1.9%)	10 (2.6%)	
**Hypertension,** yes	67 (63.8%)	96 (24.9%)	<0.001
Missing	7 (6.7%)	29 (7.5%)	
**Diabetes mellitus,** yes	55 (52.4%)	85 (22.1%)	<0.001
Missing	4 (3.8%)	31 (8.1%)	
**Heart disease,** yes	27 (25.7%)	29 (8.2%)	<0.001
Missing	5 (4.8%)	33 (8.6%)	
**Blood transfusion,** ever (yes)	12 (11.4%)	20 (5.2%)	0.03
Missing	3 (2.9%)	21 (5.5%)	

a The study included 490 participants. Information was missing for some variables in the questionnaire.

b *P* value was obtained by the chi-square test and.

c Student’s *t*-test using the complete case-analysis approach.

Hepatitis E Virus (HEV); SD = standard deviation.

A multivariable negative binomial regression model showed a-7% significant (*P* < 0.001) increase in the likelihood of HEV IgG seropositivity for each 1-year increase in age (adjusted PR1.07, 95% CI 1.04–1.09) and a significant (*P* = 0.003) association between detection of anti-HEV IgG and individuals who reported consumption of beef 2–5 times a week vs. less often: adjusted PR 2.81, 95% CI 1.40 – 5.63. Other factors such as religion, employment status, number of schooling years, consumption of seafood/clams, comorbidities, pet ownership, and blood transfusions were not significant in this model (Table 3).

**Table 3.** Multivariable negative binomial regression model of factors associated with HEV IgG seropositivity

	Adjusted prevalence ratio (95% CI)	*P* value
Age, years, continuous variable	1.07 (1.04–1.09)	<0.001
Number of schooling years	0.98 (0.90–1.07)	0.648
Religion		
Muslims	1.17 (0.45–3.01)	0.741
Druze	0.66 (0.15–2.85)	0.582
Christian	Reference	
Employment		
No	Reference	
Yes	0.81 (0.38–1.72)	0.588
Frequency of beef consumption		
2–5 times a week	2.81 (1.40–5.63)	0.003
Less than 2 times a week	Reference	
Pork consumption		
No	Reference	
Yes	0.59 (0.20–1.73)	0.343
Seafood/clams consumption		
No	Reference	
Yes	0.70 (0.27–1.81)	0.470
Pet ownership		
No	Reference	
Yes	1.23 (0.46–3.25)	0.674
Hypertension		
No	Reference	
Yes	1.56 (0.70–3.50)	0.272
Diabetes mellitus		
No	Reference	
Yes	0.66 (0.30–1.42)	0.290
Heart disease		
No	Reference	
Yes	1.33 (0.62–2.86)	0.451
Blood transfusion, ever		
Yes	0.48 (0.15–1.51)	0.21

CI: confidence interval; Included in the multivariable model were 403 participants (72 with Seropositive IgG) using the complete case analysis approach.

## Discussion

In this study, we assessed the prevalence of HEV seropositivity and related factors in the Arab population in Northern Israel. The main findings of the study are that HEV seropositivity was high in the Arab population in 2022, and it was positively related to age and frequent beef consumption. Other factors, like consuming pork or religion, as a proxy for socioeconomic status and dietary habits, were not significantly related to HEV IgG antibody seropositivity.

The overall HEV IgG seropositivity identified was 21.4%. We have previously shown a higher seroprevalence of HEV IgG antibodies in the Arab population compared to the Jewish population in Israel in 2009–2010: 22.5% vs. 10.3% [13], However, a comparison between the different population groups of Arabs, namely Muslims, Christians, and Druze, was not performed in previous studies. While these sub-population groups were shown to differ in their employment status, the number of schooling years, and also in their living environments, after controlling for these and other confounders, the multivariable analysis revealed no significant difference in HEV IgG seropositivity between these three religion groups. Our current findings demonstrate that there were no changes in the HEV seropositivity rates in the overall Arab population between the years 2010 and 2022. These rates are within the high end of the range of recently reported seroprevalence rates in European countries like France (32%), the United Kingdom (13%), and Italy (7.5%) [25], where, unlike in Israel, HEV infection causes chronic viral hepatitis. Most Israeli Arab citizens were born in Israel. Therefore, this stability in the prevalence of anti-HEV IgG antibodies, together with the age-related seroprevalence and the absence of HEV-RNA in any of the samples, suggests past exposure to the virus. Moreover, these results support the hypothesis that HEV is endemic and circulating in the country. The significant increase in the HEV IgG seropositivity with age has already been described in many studies [23], including studies from Israel [11, 13], and could result from lifetime-dependent exposure to the virus.

Frequent consumption of beef was found to be significantly (*P* < 0.001) associated with HEV IgG positivity, both in univariate and in multivariate analyses. The prevalence of HEV IgG antibody seropositivity increased by 2.81-fold in those who consume beef frequently (2–5 times a week) compared to individuals who consume beef less than two times per week. In a study that assessed risk factors for HEV in the Dutch population of blood donors, eating bovine steak and smoked beef were also positively associated with HEV seropositivity; however, this association could not be separated from the association of HEV seropositivity and eating sausages derived from pork [26]. Here, frequent beef consumption was the only dietary habit related to seropositivity and was not linked to meat derived from pigs.

In the last few years, evidence indicates that several animals other than pigs, including different ruminant species, may harbour HEV [27]. HEV RNA was found in both cow meat and cow milk [28]. To the best of our knowledge, the status of HEV in local cows, which are bred separately from pigs, in free-range beef herds, and in local dairy farms [29] has not been assessed. To enable further analysis of the association between eating beef and HEV seropositivity, data on HEV prevalence in ruminants, especially in cowsheds, dairy farms, and butcheries in Israel, should be collected.

Pig consumption, associated with being Christians, was unrelated to HEV seropositivity. These results were unexpected, as pigs in Israel were shown to be endemic for HEV-3 and as consumption of pigs, especially undercooked products, is a common risk factor associated with chronic hepatitis E. However, in Israel, swine are usually slaughtered at the age of six months, a long time after HEV infection and when only IgG against this virus can be identified [14, 15]. Together, these results may suggest that although pigs in Israel are endemic for HEV, the risk for HEV transmission from swine could probably be considered negligible.

When methods for the preparation of meat at home were assessed, most participants responded that cooking (59.4%, 291 individuals) or frying meat (29.0%, 129 individuals) were their main preparation methods for beef or pig meat. Indeed, cooking habits that may be related to HEV infection were not found to be linked to HEV IgG positivity. A thorough cooking of the pork products to an internal temperature of 71 °C or above is anyway suggested for preventing foodborne HEV infection [30].

A popular traditional dish of the Arabs, especially those located in Lebanon or Syria and also those residing in Northern Israel, is kibbeh nayeh, a national dish consumed mainly during weddings, holidays, and other social gatherings [31]. This dish is usually based on spiced red raw beef and bulgur wheat. Indeed, eating Kibbeh nayeh has already been linked to numerous food poisoning outbreaks, especially caused by microbial infections [32, 33]. Although we did not directly assess the consumption of kibbeh nayeh, the option of involvement of such traditional food warrants further research to better understand the sources and transmission modes of HEV infection in this population.

Studies that assessed other factors related to HEV seropositivity identified seafood (shellfish [34] and sea urchin [35]) as a possible factor associated with HEV infection. Herein, in the multivariable model, seafood was not significantly associated with HEV seropositivity; neither was pet ownership or clinical conditions like diabetes, heart disease, as well as blood transfusions.

Strengths of this study include the relatively large number of diverse sub-population groups of Arab participants, the high response rate (~90%), and the use of a detailed anonymous questionnaire specifically designed to assess factors related to past hepatitis E infection. An inherent limitation of such a sero-epidemiological study is that it is not accompanied with surveillance of clinical cases, and it is affected by the recall period of the questionnaire and the partial responses to some of the questions. In addition, the study population consisted of individuals living in northern Israel and might, therefore, not be fully representative of the entire Arab population. However, many Israeli Arabs reside in the north of the country, and almost all pig farms are located in that region. Moreover, the similar pattern of HEV seropositivity observed in this and previous studies in Israel is reassuring that the current results are authentic. Moreover, our study sample included individuals who attended a gastroenterology unit; their complaints included mostly gastrointestinal symptoms, abdominal pain, and heartburn, without recognized viral hepatitis or related liver diseases. Individuals who attend medical facilities might differ in their health status and lifestyle from the general population. Therefore, the generalizability of our findings might be limited. Nonetheless, the prevalence of reported health conditions and behaviours of the study participants was similar to those found in the general Arab population. Data on health status and dietary consumption was based on self-reports, which might be affected by reporting bias and social desirability. Such bias, if it exists, is expected to yield non-differential misclassification of these variables, but it is not expected to affect the measure of association in this study (prevalence ratios).

In conclusion, we found age-related seropositivity in the Arab population and identified frequent beef consumption as independent factors related to past HEV infection. Past and ongoing pork consumption were found unrelated. Studies that assess HEV seroprevalence and incidence of infection in cows, HEV and other ruminants, and provide insight on cow-farming habits on the status of HEV in cowsheds in Israel are warranted. The status of HEV in local products containing raw meat should also be assessed. In addition, the recent identification of HEV in rats and other small animals resulting in zoonotic infections [36] should also be explored.

## Supporting information

Mor et al. supplementary material Mor et al. supplementary material

## Data Availability

The original data that support the findings of this study is available as supplementary information.

## Supplementary material

For supplementary material accompanying this paper visit http://doi.org/10.1017/S0950268824001407. click here to view supplementary material
