# Supplementary material for: Hepatitis E virus (HEV) infection among the Arab population in Northern Israel: an insight into the seroepidemiology and associated risk factors
Source: Epidemiol Infect. 2025 Jan 13;153:e10. doi: 10.1017/S0950268824001407 (PMC11729521; doi:10.1017/S0950268824001407)

Epidemiology and Infection

Hepatitis E virus (HEV) infection among the Arab population in Northern Israel: An insight into the seroepidemiology and associated risk factors

Orna Mor**^*^** ^1,2^, Wasef Na'amnih ^1^, Rachel Shirazi^2^, Marina Wax^2^, Yael Gozlan^2^, Marah Kassim^1^, Helal Said Ahmad ^3^, Ali Omari ^3^, Adel Jabbour ^4^, Khitam Muhsen^#^ ^1^, Amir Mari^#3^

Supplementary Material

| **Supplementary table S1**: Descriptive statistics of HEV E IgG S/Co values in sero-negative individuals and seropositive ones | | | | | |
| --- | --- | --- | --- | --- | --- |
|  | IgG_Seropositivity IgG Results | | | Statistic | Std. Error |
| IgG_S_Co | 1 negative | Mean | | .040078605604921 | .008083569047319 |
|  |  | 95% Confidence Interval for Mean | Lower Bound | .024185007720906 |  |
|  |  |  | Upper Bound | .055972203488937 |  |
|  |  | 5% Trimmed Mean | | .011384521910838 |  |
|  |  | Median | | .005263157894737 |  |
|  |  | Variance | | .025 |  |
|  |  | Std. Deviation | | .158611078077685 |  |
|  |  | Minimum | | -.118750000000000 |  |
|  |  | Maximum | | 1.042105263157890 |  |
|  |  | Range | | 1.160855263157890 |  |
|  |  | Interquartile Range | | .036842105263159 |  |
|  |  | Skewness | | 4.150 | .124 |
|  |  | Kurtosis | | 17.986 | .248 |
|  | 2 positive | Mean | | 12.181359649122804 | .662646014104635 |
|  |  | 95% Confidence Interval for Mean | Lower Bound | 10.867307736378736 |  |
|  |  |  | Upper Bound | 13.495411561866872 |  |
|  |  | 5% Trimmed Mean | | 12.258051378446115 |  |
|  |  | Median | | 13.868421052631577 |  |
|  |  | Variance | | 46.105 |  |
|  |  | Std. Deviation | | 6.790101081789563 |  |
|  |  | Minimum | | 1.112500000000000 |  |
|  |  | Maximum | | 21.950000000000000 |  |
|  |  | Range | | 20.837500000000000 |  |
|  |  | Interquartile Range | | 12.763157894736846 |  |
|  |  | Skewness | | -.221 | .236 |
|  |  | Kurtosis | | -1.495 | .467 |

| **Supplementary table 2: comparison of reports on health conditions in the study sample vs the general Arab population** | | | | | | | |
| --- | --- | --- | --- | --- | --- | --- | --- |
| **Reports on the diagnosis of hypertension by age and sex** | | | | | | | |
| Sex | | | Hypertension | | Total |  |  |
|  |  |  | No | Yes |  | Our study | National Data - Arabs |
| Male | Age | 18-34 | 43 | 0 | 43 | 0.0% | 5.2% |
|  |  | 35-44 | 25 | 3 | 28 | 10.7% | 8.5% |
|  |  | 45-54 | 40 | 14 | 54 | 25.9% | 23.4% |
|  |  | 55-64 | 26 | 30 | 56 | 53.6% | 36.7% |
|  |  | 65-96 | 20 | 42 | 62 | 67.7% | 37.3% |
| Female | Age | 18-34 | 41 | 0 | 41 | 0.0% | 8.1% |
|  |  | 35-44 | 29 | 0 | 29 | 0.0% | 15.2% |
|  |  | 45-54 | 23 | 3 | 26 | 11.5% | 22.0% |
|  |  | 55-64 | 23 | 14 | 37 | 37.8% | 41.9% |
|  |  | 65-96 | 11 | 51 | 62 | 82.3% | 48.3% |
| **Reports on diagnosis of diabetes mellitus by age and sex** | | | | | | | |
| Sex | | | Diabetes mellitus | | Total |  |  |
|  |  |  | No | Yes |  | Our study | National Data - Arabs |
| Male | Age | 18-34 | 43 | 0 | 43 | 0.0% | 0.0% |
|  |  | 35-44 | 26 | 1 | 27 | 3.7% | 7.7% |
|  |  | 45-54 | 42 | 12 | 54 | 22.2% | 14.3% |
|  |  | 55-64 | 27 | 29 | 56 | 51.8% | 29.5% |
|  |  | 65-96 | 28 | 35 | 63 | 55.6% | 35.9% |
| Female | Age | 18-34 | 41 | 0 | 41 | 0.0% | 0.7% |
|  |  | 35-44 | 28 | 1 | 29 | 3.4% | 4.3% |
|  |  | 45-54 | 22 | 4 | 26 | 15.4% | 13.0% |
|  |  | 55-64 | 26 | 11 | 37 | 29.7% | 32.1% |
|  |  | 65-96 | 22 | 41 | 63 | 65.1% | 39.8% |
| **Reports on diagnosis of heart disease by age and sex** | | | | | | | |
| Sex | | | Heart disease | | Total |  |  |
|  |  |  | No | Yes |  | Our study | National Data - Arabs |
| Male | Age | 18-34 | 43 | 0 | 43 | 0.0% | 0.0% |
|  |  | 35-44 | 25 | 3 | 28 | 10.7% | 0.7% |
|  |  | 45-54 | 49 | 4 | 53 | 7.5% | 9.1% |
|  |  | 55-64 | 48 | 9 | 57 | 15.8% | 21.4% |
|  |  | 65-96 | 45 | 17 | 62 | 27.4% | 23.3% |
| Female | Age | 18-34 | 38 | 2 | 40 | 5.0% | 0.7% |
|  |  | 35-44 | 29 | 0 | 29 | 0.0% | 1.8% |
|  |  | 45-54 | 25 | 1 | 26 | 3.8% | 4.0% |
|  |  | 55-64 | 33 | 4 | 37 | 10.8% | 8.8% |
|  |  | 65-96 | 47 | 14 | 61 | 23.0% | 12.5% |
|  | **Physical activity** | |  |  |  | **Current smoking** | |
|  | Our data | National Data - Arabs |  |  |  | Our data | National Data - Arabs |
| Male | 27.6% | 27.0% |  |  | Male | 41.8% | 42.4% |
| Female | 18.1% | 16.5% |  |  | Female | 7.2% | 7.3% |

**Supplementary figure S1:** Histogram of the distribution of HEV E IgG S/Co values in seronegative individuals (upper panel) and seropositive ones (lower panel)


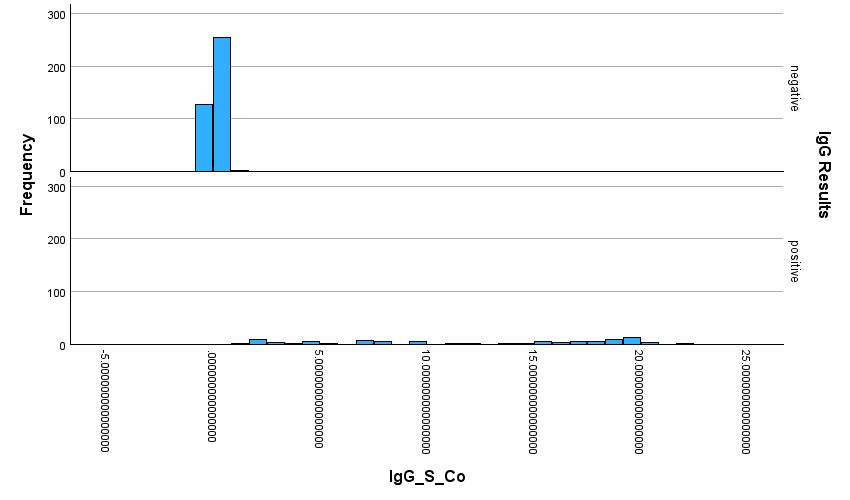


**Supplementary figure S2:** Box plot of HEV E IgG S/Co values in seronegative individuals and seropositive ones


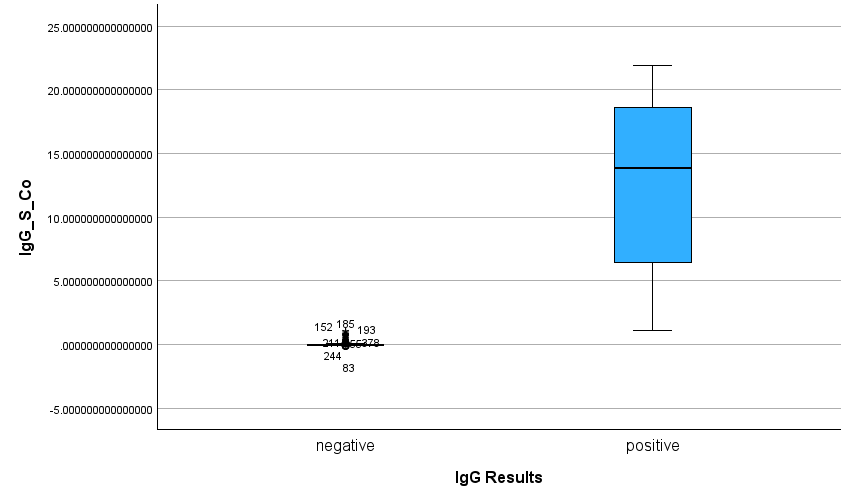

Supplement: Mor et al. supplementary material [file S0950268824001407sup001.docx]
